# Translational Physiology of Anti-Müllerian Hormone: Clinical Applications in Female Fertility Preservation and Cancer Treatment

**DOI:** 10.3389/fendo.2021.689532

**Published:** 2021-09-07

**Authors:** Rachael Jean Rodgers, Jason Anthony Abbott, Kirsty A. Walters, William Leigh Ledger

**Affiliations:** School of Women’s and Children’s Health, University of New South Wales, Sydney, NSW, Australia

**Keywords:** anti-Müllerian hormone, fertility preservation, steroid biosynthesis, aromatase, ovarian cancer, chemotherapy

## Abstract

**Background:**

Whilst the ability of AMH to induce the regression of the Müllerian ducts in the male fetus is well appreciated, AMH has additional biological actions in relation to steroid biosynthesis and ovarian follicle dynamics. An understanding of the physiology of AMH illuminates the potential therapeutic utility of AMH to protect the ovarian reserve during chemotherapy and in the treatment of female malignancies. The translation of the biological actions of AMH into clinical applications is an emerging focus of research, with promising preliminary results.

**Objective and Rationale:**

Studies indicate AMH restrains primordial follicle development, thus administration of AMH during chemotherapy may protect the ovarian reserve by preventing the mass activation of primordial follicles. As AMH induces regression of tissues expressing the AMH receptor (AMHRII), administration of AMH may inhibit growth of malignancies expressing AMHR II. This review evaluates the biological actions of AMH in females and appraises human clinical applications.

**Search Methods:**

A comprehensive search of the Medline and EMBASE databases seeking articles related to the physiological functions and therapeutic applications of AMH was conducted in July 2021. The search was limited to studies published in English.

**Outcomes:**

AMH regulates primordial follicle recruitment and moderates sex steroid production through the inhibition of transcription of enzymes in the steroid biosynthetic pathway, primarily aromatase and 17α-hydroxylase/17,20-lyase. Preliminary data indicates that administration of AMH to mice during chemotherapy conveys a degree of protection to the ovarian reserve. Administration of AMH at the time of ovarian tissue grafting has the potential to restrain uncontrolled primordial follicle growth during revascularization. Numerous studies demonstrate AMH induced regression of AMHR II expressing malignancies. As this action occurs *via* a different mechanism to traditional chemotherapeutic agents, AMH has the capacity to inhibit proliferation of chemo-resistant ovarian cancer cells and cancer stem cells.

**Wider Implications:**

To date, AMH has not been administered to humans. Data identified in this review suggests administration of AMH would be safe and well tolerated. Administration of AMH during chemotherapy may provide a synchronistic benefit to women with an AMHR II expressing malignancy, protecting the ovarian reserve whilst the cancer is treated by dual mechanisms.

## Introduction

In the late 1940s, a series of studies was conducted by Jost (1, 2) in which testosterone implants were surgically introduced into female rabbit fetuses. Whilst these experiments indicated that testosterone was capable of inducing the development of male reproductive structures including the penis and scrotum, testosterone was not shown to cause the regression of Müllerian structures. On the other hand, grafting of testicular tissue in close proximity to the ovary in female rabbit fetuses resulted in the regression of the Müllerian ducts on the ipsilateral side to the testicular tissue. This led to a proposition by Jost as to the existence of a Müllerian inhibiting substance produced by the testis (3). This substance was later identified as a testicular glycoprotein, anti-Müllerian hormone (AMH, Müllerian inhibiting substance), for which both the bovine and human genes have been isolated (1).

The AMH gene is located on chromosome 19p13.3 (4). It has five exons and four introns (1). Transcription and cleavage of this gene results in a 535 amino acid AMH protein, comprised of a 426 amino acid N-terminal prodomain and a 109 amino acid C-terminal domain that conveys the biological activity of the molecule. AMH proteins bind together *via* disulfide bonds to form homodimers, but do not become active until cleavage of the prodomain occurs (5–7). A cleavage motif exists at the arginine-serine site at residues 427-428 (5, 8–10). Mutations that block cleavage of the AMH protein at this site destroy its biological activity (10).

The prodomain is essential for correct protein folding and facilitates the dimerization of the carboxy-terminal growth factor domains. Even after cleavage of the prodomain, there is frequently a persistent non-covalent binding between the prodomain and the active protein. AMH is a member of the transforming growth factor β (TGF-β) family. This family is comprised of thirty-three members that include activins, inhibins, bone morphogenetic proteins (BMPs) and growth differentiation factors (11). In other TGF-β family members, the association of the prodomain with the active growth factor domain may inhibit binding of the active hormone to its receptor as the prodomain can form a shield over the growth factor domain (12). However the non-covalent association between the prodomain and carboxy-terminal growth factor domain of AMH that persists after cleavage greatly potentiates its activity (13), with the prodomain disassociating with the carboxy-terminal domain only after its engagement with the receptor (14).

In line with other members of the TGF-β family, AMH acts *via* a heteromeric serine/threonine receptor complex. The gene for the AMH receptor II (AMHR II) is located on chromosome 12q13 (15). The transcription of this gene results in a protein consisting of 573 amino acids. The AMHR II conveys the biological specificity of the receptor complex. It has an extracellular domain for ligand binding, a transmembrane domain and an intracellular domain with the capacity for serine/threonine kinase activity (6). A unique AMH receptor I (AMHR I) has not been identified. It appears that the function of AMHR I is conducted by a receptor that is shared with bone morphogenetic proteins (BMPs). The ALK2, ALK3 (BMPR-1A) and ALK6 (BMPR-1B) receptors have been proposed as AMHR I receptors (11, 16–19).

## Methods

The EMBASE and MEDLINE databases have been searched using search the search terms “Anti-Müllerian hormone” OR “Müllerian inhibiting substance” OR “Müllerian inhibiting hormone” OR “Müllerian inhibiting factor” in conjunction with the following terms: “physiology”, “steroid synthesis”, “fertility preservation” and “neoplasms” in combination with additional keywords associated with specific topic areas. Searches were limited to articles written in English but were not limited by date. Additional references were obtained through an analysis of the references in key articles.

## The Increasing Importance of Fertility Preservation

Globally over 8.5 million women were diagnosed with cancer in 2018. This number is projected to increase by 56.8% by 2040 to over 13.5 million women (20). Whilst the primary drivers of this increase are the growing size of the world’s population, extended life expectancy and the ageing population, even after standardization for age incidence rates have slowly increased in the last two decades. In 2017 the worldwide cancer incidence rate was 306.75 per 100,000 having risen from 296.09 per 100,000 in 1990. This is largely due to the rapid progress in preventing mortality from diseases that previously killed young people, such as infectious diseases (21).

There is a clear correlation between age and cancer incidence, with almost 80% of the new cases of cancer being diagnosed occurring in women 50 years of age or older. Despite this, a large number of pre-pubertal girls and women of reproductive age are diagnosed with cancer. An estimated 1,358,073 females aged 0-44 years were diagnosed with cancer in 2018, the most commonly diagnosed type being breast cancer, with 417,091 cases of this cancer being reported (20).

Within the 0-44 year age group, the strong correlation between cancer incidence and age is evident. Data from 2017 demonstrates an exponential rise in the number of women diagnosed with cancer in each decade of life, increasing from 272,971 cases in women aged 20-29 years, to 683,938 cases in women aged 30-39 years, and to 1,271,485 cases in women aged 40-49 years. This has important ramifications regarding the potential demand for fertility preservation as childbearing is increasingly being deferred to an older maternal age. In Australia the proportion of women having babies at less than 30 years of age halved from 80% in 1975 to 40% in 2018, with a concurrent quadrupling of the number of women aged 35 years or older giving birth, increasing from 6% in 1975 to 24% in 2018 (22). The experience of other Western countries is comparable – in England and Wales, the mean maternal age rose from 26.4 years in 1975 to 30.6 years in 2018 (23); in the United States of America, the mean maternal age at first birth rose from 21.4 years in 1970 to 26.3 in 2014 (24, 25); in the European Union, the mean maternal age rose from 29.0 years in 2001 to 30.8 years in 2018 (26).

Concurrent with these changes, cancer death rates have fallen. The worldwide age-standardized cancer death rate improved by 15% in the period 1990-2017, with a several countries including the USA, Canada, UK, Germany, France, Italy, Switzerland, Japan, Singapore and Australia reporting falls in their age-standardized cancer death rates of over 20% (27). Five-year survival rates for most cancers have also markedly improved in recent decades; in the USA for example, the overall five year survival rate rose from 50.3% in 1970-77 to 67.0% in 2007-2013.

With improving survival rates, there is an increasing focus on quality of life, of which fertility is of utmost importance. Qualitative studies consistently reiterate the importance of fertility for pre-menopausal women diagnosed with cancer (28–31). The finding that women were willing to alter their cancer treatment in order to preserve their fertility has repeatedly been reported. In a European study of 389 women aged 35 years or less at the time of breast cancer diagnosis, 8.2% of women stated that they would refuse chemotherapy if it would reduce their chance of being able to have children in the future (30); an international study of 657 women aged 40 years or less at the time of breast cancer diagnosis reported 29% of women stated that concerns regarding fertility influenced their cancer management decisions (28); an American study of 620 women aged 40 years or less at the time of breast cancer diagnosis reported that 51% of women were concerned about becoming infertile as a result of their cancer treatment, with concerns regarding fertility affecting their cancer treatment decisions in 26% of women. In this latter group of women, four women (1%) refused chemotherapy completely and 12 women (2%) altered the chemotherapy regime they would accept due to concerns regarding infertility (29).

The need for specialist involvement does not end with the instigation of cancer treatment. Fertility issues can become increasingly important to women after the initial shock of their cancer diagnosis dissipates (31). The rising incidence of cancer in pre-menopausal women and improvements in long-term survival rates, act together to increase cancer prevalence. It is estimated that the global prevalence of women aged 15-49 years with history a cancer diagnosis has increased from 14.02 million in 1990 to 24.62 million in 2017 (27). Consequently, fertility preservation strategies prior to the commencement of cancer treatment, and the ongoing management of women with a history of cancer, has transpired as a crucial component of care provided by reproductive endocrinologists. Demand for specialist care is likely to evolve in conjunction with continued advances in early cancer detection and improvements in cancer survival rates.

## The Action of AMH in the Steroid Biosynthetic Pathway

**Figure 1:**
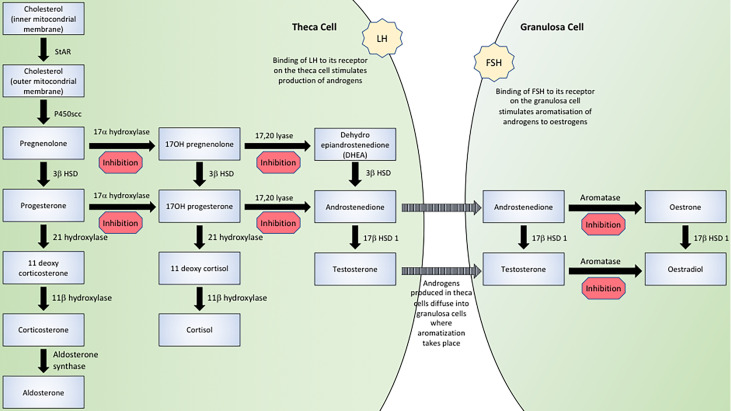
The effect of AMH in the steroid biosynthetic pathway.

AMH has several actions in the steroid biosynthetic pathway, as indicated in **Figure 1**. Whilst AMH does not suppress basal enzymatic expression, it has the capacity to mitigate hormonally mediated elevations in transcription thus acting as a regulator of sex steroid production.

The most extensively documented action of AMH in the steroid biosynthesis pathway relates to its inhibition of transcription of CYP19A1. CYP19A1 (aromatase) belongs to the cytochrome P450 superfamily and is responsible for the aromatization of androgens (androstenedione and testosterone) to oestrogens (oestrone and oestradiol respectively). *In vitro* studies of murine, porcine and human granulosa cells have demonstrated that addition of AMH to culture media does not alter the basal expression of aromatase (32–36) but does inhibit increases in aromatase mRNA transcription mediated by FSH (32–34, 36–39), LH (38) and a combination of FSH and LH (36).

FSHR and LHR are G-protein coupled receptors. The binding of FSH and LH to their respective receptors results in signal transduction modulation, at least partially through the activation of adenylyl cyclase and production of 3’5’-cycleic adenosine monophosphate (cAMP) as a second messenger (40). Thus the addition of cAMP or forskolin (a direct activator of adenylyl cyclase capable of inducing a rise in intracellular cAMP levels) mimics the action of gonadotropin downstream of FSHR and LHR. AMH has been shown to inhibit cAMP- and forskolin-mediated increase in aromatase expression (32, 35, 37–39).

As inhibition of aromatase prevents the conversion of androgens to oestrogens, inhibition of aromatase would logically be expected to decrease oestrogen and increase androgen concentrations. This has been demonstrated in one *in vitro* study in which the ovaries of sheep fetuses, obtained 29 days post-coitum, were cultured in the presence or absence of AMH, with androstenedione being used as a substrate for aromatization. Ovaries cultured in control media secreted oestradiol with only low concentrations of testosterone detected. Ovaries cultured in the presence of AMH showed a reversal of this pattern, with negligible oestradiol secretion and high testosterone concentrations detected, the latter approximating those detected in testicular culture controls. Assays of aromatase activity confirmed that the elevated testosterone and low oestradiol concentrations were secondary to reduced aromatase activity. These results were replicated by the same authors using 16-28 day post-coitum rabbit fetal ovaries (41).

Numerous *in vitro* studies report inhibition of FSH mediated increases in oestradiol by AMH with unchanged basal production of oestradiol (32, 34, 35, 38, 42). Supporting these findings is an *in vivo* study in which mice administered with 120ng or 300ng AMH for four weeks demonstrated decreased aromatase activity and decreased serum oestradiol concentrations (37).

However, the increase in testosterone production that would be expected with aromatase inhibition has not been consistently reported; conversely the majority of *in vitro* studies report *decreased* testosterone production in the presence of AMH (39, 43–46), a finding that has been replicated *in vivo* (44, 46, 47).

A study in which supra-physiological AMH concentrations in mice were generated through the administration of an adeno-associated virus serotype 9 (AAV9) vector reported decreased serum concentrations of both oestradiol and testosterone (48). Two studies of transgenic male and female mice engineered to overexpress AMH under the control of a metallothionein promoter reported significantly reduced serum testosterone concentrations in adult male transgenic mice compared to controls (47, 49); with one of these studies reporting an undetectable serum testosterone concentrations in both transgenic female mice and controls despite ovarian aromatase activity being significantly reduced in the transgenic mice, indicating testosterone does not accrue in AMH overexpressing female mice (47). A further study of male mice administered AMH *via* intra-testicular injection demonstrated decreased testicular interstitial fluid testosterone concentrations; Leydig cells obtained four hours post-injection were cultured for three hours with decreased testosterone production demonstrated in mice administered AMH (44). A final *in vivo* study in which rats were administered rAMH, reported a three-fold reduction in serum testosterone concentrations 24hrs after administration of AMH (46).

The repeated finding of decreased serum testosterone concentrations alludes to an additional inhibitory action of AMH at an earlier stage of the steroid biosynthesis pathway. Granulosa cells perform only the final stage (aromatization) of oestrogen production, the initial stages involving the conversion of cholesterol to androgens being performed in theca cells, as illustrated in **Figure 1**. Therefore *in vitro* studies of granulosa cells in which androstenedione is added to the culture media do not demonstrate a reduction in testosterone synthesis (41) as the addition of androstenedione effectively bypasses the early stages of steroid biosynthesis. *In vitro* studies in which androstenedione is not provided as a substrate (39, 43–46) and *in vivo* studies (44, 46–49), show decreased testosterone production.

Consistent with the supposition that AMH must inhibit sex steroid production at early stages of the biosynthetic pathway are findings of decreased mRNA expression of CYP17, the gene encoding 17α-hydroxylase/17,20-lyase, which is responsible for the conversion of pregnenolone/progesterone to 17OH-pregnenolone/17OH-progesterone to DHEA/androstenedione respectively (35, 43–46, 49). In an *in vitro* study, researchers cultured MA-10 cells from a Leydig cell tumour line in the presence of AMH and reported a striking ten-fold reduction in testosterone secretion after two days of culture, with a modest 40% reduction in progesterone secretion noted. Expression of CYP17 dropped to undetectable levels in the presence of AMH (45). An *in vivo* study in which adult male mice were injected with either hCG alone or hCG and AMH reported a 9-fold reduction in serum testosterone concentrations 24 hours after injection with AMH, with a slight (statistically significant) decrease in 17OH-progesterone, suggesting strong inhibition of 17,20-lyase with lesser inhibition of 17α-hydroxylase activity. Serum progesterone concentrations were unchanged (46).

Steroidogenic acute regulatory protein (StAR) delivers cholesterol from the outer to the inner mitochondrial membrane, which is the rate-limiting step in steroid biosynthesis (50). Several studies have investigated the effect of AMH on expression of StAR, with conflicting results. There are similar inconsistencies between studies that explore the impact of AMH on P450scc and 3βHSD. Some studies have reported decreased P450scc mRNA expression secondary to AMH (36, 45, 46, 49), whilst other studies report unchanged expression (36, 46). A species specific effect has been postulated after a study demonstrated a reduction in P450scc mRNA expression in rats but not in mice (46).

A final means by which AMH may influence the production of steroid hormones is by its capacity to alter hormone receptor expression. Several studies have reported either reduced expression of LH receptor (LHR) mRNA (37, 44, 49) or a blunting of an LH mediated increase in LHR expression (35). Most studies report unchanged expression of FSH receptor (FSHR) mRNA (32, 33, 37, 38) and the progesterone receptor (PR) mRNA in the presence of AMH (37). One study in which AMH was administered to mice reported unchanged androgen receptor (AR) mRNA in pre-pubertal mice and decreased AR mRNA expression in pubertal mice (37).

Observational human studies support a role of AMH in human sex steroid production with an inverse relationship between serum AMH and testosterone concentrations documented in males (51, 52) Serum AMH concentrations in males are elevated at birth and remain high until puberty when a precipitous drop occurs (51–54); serum testosterone concentrations mirror this pattern (51, 52). As AMH diminishes testosterone production due to its inhibitory action on P450 17α-hydroxylase/C_17-20_ lyase (39, 43, 45), the precipitous drop in serum AMH concentration occurring at puberty would release the inhibitory constraint of AMH on testosterone production. The decrease in AMH concentrations has been noted to occur prior to the development of clinical signs of puberty (51); a murine study has showed the pubertal increase in serum testosterone occurred 15 days after AMH concentrations decreased to basal levels (55). Also consistent with a negative correlation with testosterone concentrations, males with delayed puberty exhibit significantly elevated AMH levels, whilst males experiencing a precocious puberty have serum AMH concentrations substantially lower than those of age-matched peers (53, 56). A recent murine study has reported strong inhibitory action of testosterone and DHT on the AMH promoter (57) raising the possibility of a synergistic relationship between testosterone and AMH in males.

Data currently available based on *in vitro* cell cultures and *in vivo* animal studies indicates a strong inhibitory action of AMH on aromatase and 17α-hydroxylase/17,20-lyase, signifying that monitoring of testosterone and oestrogen concentrations would be indicated in phase I trials if AMH was administered to humans in supra-physiological amounts.

## The Role of AMH in Ovarian Follicle Dynamics

An accelerated rate of primordial follicle recruitment with premature depletion of the ovarian reserve occurs in mice engineered to be homozygous or heterozygous for an inactivating mutation of the AMH gene (58–61). In one study, it was reported that prior to puberty (25 days of age), primordial follicle numbers were comparable between AMH deficient and wild type mice (60). During puberty (four months of age), a more rapid recruitment of primordial follicles was demonstrated, with increased numbers of pre-antral and antral follicles in mice carrying the loss-of-function AMH gene mutation. Towards the end of the reproductive lifespan (thirteen months of age), an almost complete depletion of the primordial follicle pool in mice homozygous for the AMH mutation had occurred, with a lesser reduction exhibited in heterozygous mice. Consistent with a premature exhaustion of the ovarian reserve, a subsequent study reported that 56% of AMH knockout mice had ceased ovulation by 16-17 months of age, compared to 18% of wild-type mice (62).

Short-term *in vitro* cultures of both murine and human ovarian tissue have documented results consistent with AMH acting to restrain primordial follicle development. The ovaries of two-day old mice, when cultured in the presence of AMH were found to contain 59% less primary and secondary follicles compared to control ovaries (p≤0.05), consistent with AMH restraining primordial follicle recruitment; after four days of culture this difference had widened to 66% (p≤0.05) (58). To investigate this effect in humans, ovarian tissue obtained from women aged 26-42 years (mean 33.7+/-3.6 years) and was cultured in the presence or absence of AMH. In uncultured control samples, 56% of follicles were observed to be at the primordial follicle stage; after seven days of culture, the proportion of follicles at primordial follicle stage had decreased to 14-26% in specimens cultured with 0-30ng/mL AMH, (p≤0.05). When cultured in the presence of 100ng/mL, there was no significant difference in the proportion of primordial follicles compared to uncultured ovarian tissue specimens (63).

An extended tissue culture (four weeks) of human ovarian tissue has not replicated this inhibitory action of AMH in primordial follicle recruitment. In uncultured ovarian tissue, 90.5% of follicles were found to be at the primordial follicle stage compared to 54%, 46%, 35% and 43% in samples cultured in control media or media supplemented with testosterone, AMH or both AMH and testosterone respectively (p≤0.05 for all groups) (64). The divergent findings are possibly explained by the prolonged period of tissue culture or by the culture media used in this study containing α-MEM, which has been demonstrated to result in significantly greater follicle initiation and growth compared to other culture media (65).

There is a limited amount of data to suggest that AMH may have a supportive role in later stages of follicular growth. In one study, pre-antral follicles (140-150μm) were dissected from ovaries obtained from 12 day old rats and were individually cultured in wells containing FSH, AMH or both FSH and AMH (66). After 72 hours, there was no significant growth of follicles cultured in control media. Follicles increased in size by 10μm when cultured in the presence of AMH, by 25μm in the presence of FSH and by 40μm in the presence of both FSH and AMH, suggesting a facilitatory role of AMH on secondary follicle growth.

In a primate study of adult female macaques, AMH was determined to promote the formation of an antrum in growing follicles (67). In this study, secondary follicles (diameter 125-225μm, 2-4 layers of granulosa cells) were isolated and cultured in individual wells for five weeks. AMH or a neutralizing anti-AMH antibody was added to the wells. Follicles developed an antrum earlier when AMH was added to the culture media in weeks 0-3; the addition of the anti-AMH antibody delayed antrum formation. Oestradiol production was markedly low at week 5 in follicles exposed to AMH compared to control follicles, consistent with an inhibitory effect of AMH on oestradiol production.

In the second part of this study, intra-ovarian infusions of either control of 500ng/hour of anti-human AMH antibodies were administered to adult macaques for day 1-4 of the menstrual cycle until the mid-cycle oestradiol peak (67). In the macaques administered anti-AMH antibodies, antrum formation was delayed. Based on this data, the authors have submitted that AMH facilitates the pre-antral to antral development of ovarian follicles in primates.

## Mechanisms of Chemotherapeutic Damage to the Ovaries

Mechanisms by which chemotherapy may cause depletion of the ovarian reserve include a detrimental effect to the stroma or vasculature; direct damage to the oocytes inducing apoptosis; damage to the granulosa cells that comprise the follicles containing the oocytes; or unrestrained activation of primordial follicles with their consequent rapid loss from the ovarian reserve (68). There is evidence supporting each of these hypotheses.

Vascular damage is an established consequence of many chemotherapeutic agents as demonstrated in a trial in which human ovarian tissue was either cultured with doxorubicin for 72 hours, or xenografted into SCID mice that were then treated with doxorubicin. Reduced vascular density after exposure to doxorubicin was reported in both the *in vitro* and *in vivo* studies (69). In a human study, ovarian tissue samples were obtained from 35 women with cancer (mean age 28.7+/- 7.74 years) of whom 17 had been exposed to non-sterilizing chemotherapy prior to laparoscopic harvest of ovarian tissue. In a blinded histopathological examination of specimens, thickening and hyalinization of large stromal vessels, disordered neovacularisation and regions of focal fibrosis was identified in the ovarian cortex specimens obtained from women who had received chemotherapy (70). Concurring with these findings is a study of ovarian tissue biopsies obtained from girls who had successfully completed chemotherapy treatment for acute lymphoblastic leukaemia; narrowed capillaries with irregular lumens and pericapillary stromal fibrosis was observed in these specimens (71). Whilst these studies establish a feasible mechanism in which chemotherapy causes microvascular damage to the ovarian cortex, potentially leading to areas of localized ischaemia and resulting in primordial follicle loss, causality has not been proven.

The hypothesis that damage to the ovarian reserve is due to direct cellular damage to the oocyte is well founded (68). A study in which high dose cyclophosphamide was administered to mice demonstrated disruption to the morphology of the oocyte with the nuclear contents becoming clumped and distorted within 24-72 hours of administration. The surrounding granulosa cells appeared unaffected upon histological analysis. Complete destruction of the oocyte followed, leaving an empty ring of granulosa cells (72). Another study has demonstrated doxorubicin induces double-strand DNA breaks in both oocytes and granulosa cells (69).

One mechanism of action of alkylating agents, such as cyclophosphamide, is to induce abnormal bonds, or cross-links, between DNA bases preventing strand separation required for transcription or translation. Administration of cyclophosphamide to female rats primed with pregnant mare serum gonadotrophin reported an increase in DNA cross-linkage in granulosa cells two hours after administration. Twenty-four post-administration, granulosa cell numbers were depleted by 51% (73), indicating that damage to the ovarian reserve caused by chemotherapy is not confined solely to direct damage to oocytes. Consistent with this finding was a study of female mice administered with cyclophosphamide that used TUNEL (terminal deoxynucleotidyl transferase dUTP nick end labelling) to detect DNA fragmentation and apoptosis which demonstrated a dose dependent increase in apoptosis of the granulosa cells of preantral and antral follicles. This effect did not extend to the granulosa cells of primordial or primary follicles. This finding is likely explained by the higher mitotic index of the granulosa cells of larger follicles, rendering them more sensitive to cyclophosphamide (74).

Another hypothesis regarding the mechanism of chemotherapeutic damage is that a sharp drop in serum oestradiol concentrations occurs upon the commencement of chemotherapy, with the consequent decrease in the negative inhibitory action on the anterior pituitary causing an elevation in serum FSH concentrations. The FSH elevation would be expected to cause an upsurge in the rate of granulosa cell proliferation, increasing the susceptibility of these cells to the anti-proliferative and cytotoxic effects of chemotherapy. With continued follicular destruction and further FSH elevation, more follicles are recruited and destroyed, eventually resulting in primordial follicle depletion (75). If this theory is correct, medications that inhibit the release of FSH, namely GnRH agonists, administered during chemotherapy should be protective, however studies in this area have shown divergent results (76–79). Additionally, a study of the hormonal sequalae of chemotherapy has not shown a dramatic drop in serum oestradiol concentrations immediately upon the commencement of CMF (cyclophosphamide, methotrexate, 5-fluorouracil) treatment (80). Further arguing against this theory, is that the finding that granulosa cells of primordial follicles do not express FSH receptor mRNA (81), indicating that primordial follicle recruitment must take place independently of FSH.

Despite these flaws, this hypothesis has merit in suggesting that ‘burn out’ of the primordial follicle reserve may be an important mechanism by which chemotherapy provokes destruction of the ovarian reserve. In a murine study, increasing doses of cyclophosphamide have been shown to cause an upsurge in the ratio of early growing follicles compared to dormant follicles (82). Should a means of restraining primordial follicle activation become available, its use during chemotherapy may be protective to the ovarian reserve.

## Ability of AMH to Protect the Ovaries During Chemotherapy

If AMH is able to restrain primordial follicle activation, it may be able to offer a degree of protection to the ovarian reserve during chemotherapy. To evaluate this hypothesis, carboplatin (80mg/kg IP) or doxorubicin (3mg/kg IP) was administered weekly to tumour (ovarian cancer) bearing Nu/Nu mice. AMH concentrations were maintained through the use of an AAV9-AMH viral vector which had previously been demonstrated to convey a sustained elevation in serum AMH concentrations. Mice were euthanized when tumour-related end points were met. In the presence of the AMH viral vector, primordial follicle counts were reported as being 2.2-fold higher in mice receiving carboplatin and 1.8-fold higher in mice receiving doxorubicin, compared to controls receiving chemotherapy alone. In a subsequent experiment, osmotic pumps containing an rAMH solution were implanted in mice. Weekly chemotherapy with carboplatin, doxorubicin or cyclophosphamide was then instituted, with the euthanized after two weeks of chemotherapy. Primordial follicle counts were higher in the mice implanted with the rAMH osmotic pumps (1.4-fold higher for carboplatin-treated mice, p<0.0001; 2.9-fold higher in doxorubicin-treated mice, p<0.001; 1.2-fold higher in cyclophosphamide-treated mice, p<0.05) compared to mice implanted with saline pumps (48).

Another study involved the administration of a single intraperitoneal injection of either vehicle, 150mg/kg cyclophosphamide, 5mg/kg rAMH or both cyclophosphamide and rAMH to female mice before being euthanized either 17 hours or 8 days later. The primordial follicle count was reduced in the cyclophosphamide treated mice compared to the control mice (448.2+/-55.0 versus 1197+/-138.2 follicles, p<0.01), whereas the number of primordial follicles in the ovaries of mice receiving both cyclophosphamide and rAMH was similar to that of controls (813.4+/-68.7 versus 875.5+/-83.9, p=NS) (83).

The protective effect of AMH on the ovarian reserve during chemotherapy has been demonstrated to translate into an improved fertility outcome in one of two studies. The authors of the study demonstrating a protective effect of AMH (84) administered two doses of cyclophosphamide (150mg/kg) to mice either in isolation or in conjunction with rAMH (four doses of rAMH administered six hourly following each dose of cyclophosphamide). Mice were then mated with males of proven fertility six successive times commencing five weeks after the final dose of chemotherapy. Mice exposed to cyclophosphamide alone demonstrated a reduced pregnancy rate and a smaller mean litter size compared to controls (pregnancy rate: 0.26+/-0.07 versus 0.50+/-0.06, p<0.05; mean litter size 1.2+/-0.3 versus 3.0+/-0.6). Mice exposed to chemotherapy plus AMH had a similar pregnancy rate to controls (0.54+/-0.06 versus 0.50+/-0.06), with a higher mean litter size compared to mice that received cyclophosphamide alone (2.3+/-0.4 versus 1.2+/-0.3).

The second study (83) involved the weekly intraperitoneal administration of either vehicle or a non-sterilizing dose (75mg/kg) of cyclophosphamide for four weeks, either in isolation or in combination with rAMH. Four weeks after the final injection, mating with males of proven fertility took place. The authors reported a non-significant reduction in the cumulative number of mice born in the group receiving cyclophosphamide alone compared to control mice and mice receiving both cyclophosphamide and rAMH (47.3+/-5.9 versus 53.2+/-8.2 versus 53.5+/-10.5). The lack of statistical effect demonstrated may be due to the lower dose of cyclophosphamide (75mg/kg) used in this study, compared to dose (150mg/kg) used in the study that demonstrated a protective effect on litter size with AMH administration.

The administration of AMH during chemotherapy must be conducted with care, avoiding the abrupt withdrawal of exogenous AMH. In a normally functioning ovary, follicles at all stages (primordial, primary, secondary, antral) coexist at the same time. When high levels of exogenous AMH are administered, there is a marked reduction in the number of primary, secondary and antral follicles (48). As the granulosa cells of the secondary, pre-antral and early antral follicles secrete AMH (85), a drop in these follicle numbers would be expected to make the ovary unable to continue to secrete AMH should the administration of exogenous AMH be abruptly ceased. This notion is supported by an observation that immediately post AMH cessation, there is a large and rapid loss of primordial follicles (48).

## Role in Ovarian Tissue Transplantation

The potential use of AMH in respect to fertility preservation is not limited to its administration during chemotherapy. In pre-pubertal girls, or women for whom oocyte cryopreservation is not an option prior to the instigation of gonadotoxic treatment, ovarian tissue may be cryopreserved prior to the instigation of treatment, with regrafting of the ovarian tissue after successful cancer therapy. Whilst over 130 live births have occurred as a result of this technology (86), there is a massive follicular loss immediately post-transplantation secondary to ischaemia and rapid primordial follicle activation (87–89). This has resulted in a high denominator of transplantation attempts to achieve the reported number of live births. One case series of 111 women undergoing transplantation of cryopreserved ovarian cortex reported that 32 women (29%) conceived and 23 women (21%) had a live birth (a total of 33 live births, with five women delivering more than once and two twin deliveries) (90). Another case series of 95 orthotopic transplantations in 74 women, reported that 16 women conceived (17%) of these women, 15 continued to a live birth (16%) (a total of 17 live births with two women delivering twice) (91).

The transplantation of ovarian cortical tissue into women who have undergone sterilizing cancer treatment (and thus have no ovarian function) introduces the ovarian tissue into an environment devoid of AMH. Parallels can be drawn to the culture of ovarian cortical tissue in media that does not contain AMH; *in vitro* studies consistently demonstrate rapid primordial follicle activation in this situation. One study that utilized ovarian cortex sourced from cattle and baboons demonstrated that the majority or primordial follicles were activated within 12-24 hours of culture in AMH-free media (92). In a study of bovine ovarian cortex, 72% of follicles in freshly harvested tissue were at the primordial stage of development, within two days of culture only 10% remained at the primordial follicle stage (93). In a quantitative analysis of ovarian tissue specimens obtained from women aged 25-42 years (mean age 35 years) who underwent oophorectomy, 88% of follicles were reported to be at the primordial stage, with 8% at the primary stage when examined immediately post-oophorectomy; after 4-11 days of culture, only 20% of follicles were reported to be at the primordial follicle stage, with 65% at the primary stage after 4-11 days (94).

The results of an *in vivo* studies in which human ovarian tissue was grafted into ovarectomized (SCID) mice are consistent with the *in vitro* studies. The proportion of primordial follicles present in the ovarian tissue fell from 72.88+/-5.93% in pre-graft control specimens to 38.95+/-3.94% four weeks after grafting (p<0.001) and 37.42+/-5.83% (p=0.009) twelve weeks after grafting. There was a concomitant rise in the percentage of primary follicles from 13.48+/-2.92% in pre-graft control specimens, to 29.29+/-2.60% four weeks after grafting (p=0.009), but twelve weeks after grafting the elevation in primary follicle percentages was not sustained, with 9.74+/-2.22% of follicles found to be at the primary follicle stage. This latter result is likely due to a substantial number of the primary follicles progressing to the secondary stage [pre-graft versus four week graft 13.37+/-6.09% versus 27.60+/-4.16% (p=0.018); pre-graft versus twelve week graft 13.37+/-6.09% versus 49.40+/-6.14 (p=0.001)] (95). These results concur with a similar study that reported 71% of follicles were at the primordial stage in fresh human ovarian cortex; one week after grafting into nude mice, this proportion had decreased to 39% (p<0.001); a concurrent rise in the proportion of primary follicles from 11% in fresh tissue to 24% stage one week after grafting was observed (p=0.05) (87).

The mass activation of primordial follicles that occurs in the absence of AMH is in contrast to the orderly activation of primordial follicles that occurs in the ovaries of healthy women. A key difference between these two situations is that AMH is present in the circulation of the healthy women. The pivotal role that AMH has in controlling primordial follicle activation was demonstrated in a study in which human ovarian cortex was cultured in the presence or absence of AMH. Rapid depletion of the primordial follicle pool occurred in AMH-free media, however when 100ng/mL AMH was added to the media, the proportion of follicles maintained at the primordial stage was comparable to that of uncultured tissue (63). Another study using bovine ovarian cortex demonstrated that when this tissue was cultured in serum-free media, a seven-fold decrease in primordial follicles occurred. When bovine ovarian cortex was cultured on the edge of the chorioallantoic membrane (CAM) of chick embryos, primordial follicle numbers were unchanged from day 0, indicating a component in the environment of the ovarian tissue was able to restrain the activation of primordial follicles (92).

The clinical experience of human auto-transplantation of cryopreserved ovarian cortex is consistent with unrestrained primordial follicle activation. In a longitudinal analysis of eleven young menopausal recipients of fresh ovarian cortex transplants (donated by an identical twin, n=9, or a non-identical sibling, n=2) AMH concentrations initially remained low before sharply rising to supra-physiological levels (at approximately 170 days post grafting), consistent with a rapid progression of primordial follicles to the secondary stage whereupon AMH secretion is commenced. Shortly thereafter, a sustained fall to below to sub-physiological concentrations was observed (approximately 240 days post grafting), consistent with a decrease in secondary follicles secondary to depletion of the primordial follicle pool (96).

It is conceivable that the administration of exogeneous AMH could temper the rapid initial recruitment and subsequent loss of primordial follicles until reperfusion of the graft has taken place and intrinsic AMH production commenced. To elucidate the effect of AMH on ovarian tissue, researchers cryopreserved murine ovarian tissue in vitrification media containing different concentrations of human AMH (0, 5, 15 or 45μg/mL) before warming the tissue in media using the same concentrations of AMH. No difference was detected in the proportion of primordial, growing or grade 1 follicles between the different dose groups of AMH, although there was a reduction in the proportion of apoptotic follicles from 21.0% in the control group compared to 8.1% in the 5μg/mL AMH group and 1.7% in the 15μg/mL and 45μg/mL AMH groups (97). The lack of difference in the proportion of follicles at different stages may be explained by short duration (total 35 minutes) of exposure of the ovarian tissue to AMH.

The same researchers then vitrified fresh ovarian cortex in media devoid of AMH, before warming and auto-transplanting the tissue back into the mice one week later. The mice were divided into groups and received either 0, 50, 250 or 1,250μg/mL human AMH doses every two days for a total of four doses prior to ovariectomy, immediately post ovariectomy or both pre- and post-ovariectomy. Mice were euthanized either 7 or 28 days after auto-transplantation. No statistical difference in the proportion of primordial or growing follicles was identified in any of the treatment groups (97). The absence of effect in the second study may be explained by an insufficient frequency of AMH. The decision of the authors to administer AMH every two days was based on a report estimating the half-life of bovine AMH to be approximately 48 hours (98), however a study of human AMH has estimated the half-life as 27.6 hours (99).

Further *in vivo* research using human ovarian cortex is required to clarify whether AMH has the capacity to curtail uncontrolled mass activation of primordial follicles in the immediate post-transplantation period. The prolonged timeframe between fresh ovarian tissue grafting in young menopausal women and rise in AMH (96), suggests an extended duration of AMH administration may be necessary.

## Role of AMH in the Treatment of Cancer

As its name suggests, the renowned physiological function of AMH is to induce the regression of the Müllerian ducts in the male fetus. It has been hypothesized, and successfully demonstrated, that this inhibitory action can be utilized to induce the regression of malignancies expressing AMHRII (100). Studies have demonstrated that AMH is capable of inhibiting the proliferation of cancers arising from Mullerian structures such as the cervix (101, 102) and endometrium (103, 104), as well as some cancers arising from non-Mullerian structures that express AMHR II such as breast (105–107), vulva (108) and prostate (106). The main focus of investigation however has been in regard to ovarian cancer. Despite the ovaries not being Mullerian structures, there is increasing concurrence that many ovarian neoplasms are of Mullerian origin (109–111), explaining why AMHR II receptors are expressed by many ovarian cancer cells (112, 113).

Initial studies used partially purified bovine AMH to demonstrate that AMH was capable of inhibiting growth *in vitro* of human papillary serous cystadenocarcinoma cells (100). *In vivo*, pretreatment of ovarian cancer cells (HOC-21 cell line) with bovine AMH delayed the appearance of tumour and increased disease free survival when these cells were subcutaneously injected into the middorsal flank of Balb/C nude mice (114). Fresh tumour suspensions were obtained from twenty-eight women undergoing surgery for gynaecological cancer (ovarian, endometrial or fallopian tube origin) and were tested in soft agar colony inhibition assays. Significant colony inhibition was demonstrated in 25 of 28 assays following incubation with bovine AMH (115).

After the human AMH gene was isolated, it was transfected into Chinese hamster ovary cells to provide a more purified form of AMH (recombinant AMH, rAMH). *In vitro* studies confirmed that rAMH could inhibit both human and murine ovarian cancer cell lines (112, 113, 116, 117). In one study, six human ovarian cancer cell lines expressing AMHR II were incubated in the presence of 15ug/mL rAMH (113). rAMH caused almost complete inhibition of growth in two cell lines, and significant inhibition of growth in three other lines. In the final cell line colony growth was not inhibited, possibly secondary to impaired downstream signaling due to an absence of functional p16. Ascites was then obtained from 27 women with ovarian cancer of which 15 (56%) contained malignant cells that bound biotinylated AMH, suggesting expression of AMHR II. Out of the eleven cell lines that bound biotin and grew in soft agarose, 9 of 11 (82%) were significantly inhibited (29-94% inhibition) when rAMH was added to the culture medium.

*In vivo* studies using murine (MOVCAR7, MOVCAR8) and human (OVCAR3, OVCAR8, IGROV-1) cancer cell lines injected into immunodeficient mice have also demonstrated an inhibitory effect of rAMH (108, 117, 118). Additionally, rAMH may have anti-metastatic action, having been shown to decrease invasiveness in an *in vitro* study using the epithelial ovarian cancer cell line IGROV-1, and inhibit migration in an *in vivo* study using a chick chorioallantoic membrane migration assay (119).

Epithelial ovarian cancer is a highly lethal cancer due to an advanced disease stage at the time of diagnosis in the majority of cases and chemo-resistance that may be intrinsic or develop with disease progression (120). High grade ovarian cancers exhibit biological features, including molecular heterogeneity, the capacity to metastasize and an ability to develop chemo-resistance, that support the proposal that it is a cancer stem cell driven disease. Cancer stem cells are capable of unlimited self-renewal and have an ability to differentiate through asymmetric cell division. As they are pluripotent, they can give rise to daughter cells with different phenotypes, permitting tumour heterogeneity and chemo-resistance (121). Their relatively quiescent state and expression of proteins capable of acting as molecular pumps, effluxing lipophilic medications out of the cell, permit these cells to evade destruction by chemotherapeutic and radiation treatments (121, 122).

As AMH acts by binding to the extracellular AMHR II rather than by diffusing into cells, it has the capacity to act on chemo-resistant cancer stem cells. An *in vitro* study demonstrated that whilst treatment of cells from the OVCAR-5 ovarian cancer cell line with doxorubicin, cisplatin and paclitaxel resulted in a decrease in the total number of viable cells, there was an expansion in the proportion of cells exhibiting stem cell characteristics. Treatment with AMH caused a significant decrease in both the total number of cells and in the stem cell population (123). An additional study that isolated stem cell enriched populations of ovarian cancer cell lines concurred that doxorubicin treatment stimulated the growth of these cells, whilst AMH inhibited proliferation by inducing G1 arrest through the induction of cyclin-independent kinase inhibitors (124).

The potential clinical utility of AMH to inhibit the proliferation of chemo-resistant ovarian cancer cells was demonstrated in a study in which malignant cells were obtained from the ascites of women with highly resistant ovarian cancer. *In vitro*, four of six cell lines were inhibited when cultured in media containing AMH. To investigate *in vivo* action, five patient-derived lethal chemo-resistant serous adenocarcinoma lines were xenografted into mice. AMH administration inhibited the proliferation of three of the five tumours (125).

AMH also has potential utility in immunotherapy-based approaches to cancers expressing AMHR II. A mouse antibody (12G4) has been developed to bind to the human AMHR II. When administered to nude mice xenografted with human granulosa cell (COV434) or epithelial ovarian cancer (OVOCAR-3) cell lines, tumour growth was inhibited *via* antibody-dependent cell-mediated cytotoxicity (possible as nude mice have functional macrophages and natural killer cells) and, to a lesser extent, due to the activation of signaling pathways after receptor/ligand complex internalization (126).

Murlentamab (GM102 or 3C23K) is a human monoclonal antibody designed to bind to the AMHRII expressed by malignant cells. The Fc portion of the antibody has been glycol-engineered to have low fucosylation, enabling high affinity binding to CD16 and thus enhancing natural killer cell activity as well optimizing antibody-dependent cell-mediated cytotoxicity (ADCC) and antibody-dependent cellular phagocytosis (ADCP) (127, 128). A phase I trial of murlentamab of 68 women with metastatic AMHRII-expressing ovarian, cervical or endometrial cancer who had previously been treated with at least one chemotherapy regime reported that the antibody was well tolerated with no dose limiting toxicity; the most common adverse effect was G 1-2 asthenia (29%); a lesser number of patents (12%) reported more severe asthenia, nausea or vomiting (129). Approximately 80% of colorectal adenocarcinomas express the AMHRII, consequently a phase II study of the efficacy of murlentamab in patients with advanced colorectal adenocarcinoma has been conducted (130). Thirty nine patients received either murlentamab alone (n=14) or in conjunction with trifluridine and tipiracil (n=15), with the study reporting a 1.7-fold and a 3.6-fold reduction in the tumour growth rate in these groups respectively. Of fourteen patients treated with murlentamab alone, those with greater than 20% AMHRII positive tumour cells, showed superior rates of progression-free survival.

Antibodies specific for AMHR II have been radiolabelled with 213Bi to effect destruction of AMHR II expressing intra-peritoneal tumours in mice (131) and can be used as a means of targeted drug delivery whereby cytotoxic drugs are covalently attached to AMH. Upon internalization, the bonds between AMH and the cytotoxic molecule are cleaved by proteases, freeing the drug to accomplish its cytotoxic function (9, 121). The use of radiolabelled diabodies (constructed from fragments of antibodies capable of binding to AMHR II), has been proposed as a new immunoimaging diagnostic and monitoring approach to gynaecologic malignancies (132).

AMH in the treatment of cancer has a number of potential advantages over traditional chemotherapeutic agents. Firstly, increased concentrations of AMH do not convey the severe toxicity associated with many chemotherapeutic medications. AMH concentrations of up to 480ng/mL in women with PCOS (133) and 1,200ng/mL in women with granulosa cell tumours (134) have been reported without evidence of adverse effects. Secondly, AMH acts *via* a different mechanism to traditional chemotherapeutic agents and therefore may be effective in the treatment of cancer stem cells and chemo-resistant cancers. Finally, in contrast to many chemotherapy medications currently used in clinical practice that damage the ovaries and thus reduce the likelihood of future fertility, AMH would not be expected to cause a depletion of the ovarian reserve provided its administration is not abruptly ceased. If AMH was co-administered during chemotherapy, it may have a synchronous action to the chemotherapy, and may also offer a degree of protection to the ovaries.

## Conclusions and Future Directions

Preliminary animal data indicates that AMH has clinical application in protecting the ovarian reserve during chemotherapy, suppressing primordial follicle recruitment whilst vascularization of ovarian tissue grafts is re-established and in the treatment of malignancies expressing AMHR II.

To date, AMH has not been administered to humans. A comprehensive review of the biological actions of AMH suggests that its administration would be safe and well tolerated, although a decrease in sex steroid production would be expected due to its inhibitory of aromatase and 17α-hydroxylase/17,20-lyase. Depending on the degree of suppression, oestradiol supplementation may be required to avoid menopausal symptoms or if AMH administration was prolonged. Administration of an AMHRII antibody (murlentamab) has been well tolerated in a small number of cancer patients (129, 130).

Epithelial ovarian cancer accounts for approximately 90% of ovarian cancer diagnoses, of which the majority have serous tumour cell histology. 80% of serous epithelial ovarian cancers are diagnosed at an advanced (III-IV) stage. The overall five year life expectancy of epithelial ovarian cancer in the USA in 2007-13 was 41% for women diagnosed at stage III and 20% for women diagnosed at stage IV (135) as chemo-resistance inevitable occurs leaving few treatment options aside from palliation. In light of these grim statistics, human trials of AMH administration in women with advanced ovarian cancer would be appropriate, especially data suggests that AMH can be effective in inducing the regression of chemo-resistant tumour cells.

Additional animal studies are required to confirm and detail the value of AMH in fertility preservation agent, both in its capacity to protect the ovarian reserve during chemotherapy and in the context of ovarian tissue grafting after successful cancer treatment. If data consistently confirms the beneficial denoted in preliminary studies, a trial of adjuvant AMH during chemotherapy in women of reproductive age would be pertinent.

As the administration of AMH would be expected to suppress primordial follicle recruitment and development, extended administration of AMH is likely to decrease the number of pre-antral follicles present in the ovaries, with a consequent decrease in intrinsic AMH secretion. Abrupt cessation of AMH administration would therefore be expected to result in a transient period of sub-physiological AMH serum concentrations and uncontrolled primordial follicle recruitment could occur. This mandates caution with use of AMH for the purpose of preserving the ovarian reserve during chemotherapy; graduated dose reduction prior to cessation is warranted.

## Author Contributions

RR conceived and designed the article, conducted the review of the literature, interpreted the data and wrote the manuscript. JA contributed to data analysis and conducted a critical revision of the article. KW conducted a critical revision of the article. WL contributed to data analysis and conducted a critical revision of the article. All authors contributed to the article and approved the submitted version.

## Conflict of Interest

The authors declare that the research was conducted in the absence of any commercial or financial relationships that could be construed as a potential conflict of interest.

## Publisher’s Note

All claims expressed in this article are solely those of the authors and do not necessarily represent those of their affiliated organizations, or those of the publisher, the editors and the reviewers. Any product that may be evaluated in this article, or claim that may be made by its manufacturer, is not guaranteed or endorsed by the publisher.

## References

[1] Cate RL Mattaliano RJ Hession C Tizard R Farber NM Frey AZ. Isolation of the Bovine and Human Genes for Mullerian Inhibiting Substance and Expression of the Human Gene in Animal Cells. Cell (1986) 45(5):685–98. 10.1016/0092-8674(86)90783-X 3754790

[2] Price JM Donahoe PK Ito Y Hendren WH. Programmed Cell Death in the Mullerian Duct Induced by Mullerian Inhibiting Substance. Am J Anat (1977) 149:353–76. 10.1002/aja.1001490304 879051

[3] Jost A. Problems of Fetal Endocrinology: The Gonadal and Hypophyseal Hormones. Recent Prog Hormone Res (1953) 8:379–413.

[4] Cohen-Haguenauer O Picard JY Mattei MG Serero S van Cong N de Tand MF. Mapping of the Gene for Anti-Mullerian Hormone to the Short Arm of Human Chromosome 19. Cytogenet Cell Genet (1987) 44(1):2–6. 10.1159/000132332 3028714

[5] Belville C Van Vlijmen H Ehrenfels C Pepinsky B Rezaie AR Picard JY. Mutations of the Anti-Mullerian Hormone Gene in Patients With Persistent Mullerian Duct Syndrome: Biosynthesis, Secretion, and Processing of the Abnormal Proteins and Analysis Using a Three-Dimensional Model. Mol Endocrinol (2004) 18(3):708–21. 10.1210/me.2003-0358 14673134

[6] Josso N di Clemente N. Transduction Pathway of Anti-Müllerian Hormone, a Sex-Specific Member of the TGF-β Family. Trends Endocrinol Metab (2003) 14(2):91–7. 10.1016/S1043-2760(03)00005-5 12591180

[7] Josso N di Clemente N Gouedard L. Anti-Mullerian Hormone and its Receptors. Mol Cell Endocrinol (2001) 179:25–32. 10.1016/S0303-7207(01)00467-1 11420127

[8] Kurian MS de la Cuesta RS Warneck GL MacLaughlin DT Manganaro TF Donahoe PK. Cleavage of Mullerian Inhibiting Substance Activates Antiproliferative Effects *In Vivo* . Clin Cancer Res (1995) 1:343–8. 9815990

[9] MacLaughlin DT Donahoe PK. Mullerian Inhibiting Substance/Anti-Mullerian Hormone: A Potential Therapeutic Agent for Human Ovarian and Other Cancers. Future Oncol (2010) 6(3):391–405. 10.2217/fon.09.172 20222796 PMC3935316

[10] Nachtigal MW Ingraham HA. Bioactivation of Mullerian Inhibiting Substance During Gonadal Development by a Kex2 / Subtilisin-Like Endoprotease. Proc Natl Acad Sci (1996) 93:7711–6. 10.1073/pnas.93.15.7711 PMC38812 8755541

[11] Visser JA Olaso R Verhoef-Post M Kramer P Themmen AP Ingraham HA. The Serine/Threonine Transmembrane Receptor ALK2 Mediates Mullerian Inhibiting Substance Signalling. Mol Endocrinol (2001) 15(6):936–45. 10.1210/mend.15.6.0645 11376112

[12] Shi M Zhu J Wang R Chen X Mi L Walz T. Latent TGF-Beta Structure and Activation. Nature (2011) 474(7351):343–9. 10.1038/nature10152 PMC4717672 21677751

[13] Wilson CA di Clemente N Ehrenfels C Pepinsky RB Josso N Vigier B. Mullerian Inhibiting Substance Requires its N-Terminal Domain for Maintenance of Biological Activity a Novel Finding With the Transforming Growth Factor-B. Mol Endocrinol (1993) 7(2):247–57. 10.1210/mend.7.2.8469238 8469238

[14] di Clemente N Jamin SP Lugovskoy A Carmillo P Ehrenfels C Picard JY. Processing of Anti-Mullerian Hormone Regulates Receptor Activation by a Mechanism Distinct From TGF-Beta. Mol Endocrinol (2010) 24(11):2193–206. 10.1210/me.2010-0273 PMC5417381 20861221

[15] Imbeaud S Faure E Lamarre I Mattei MG di Clemente N Tizard R. Insensitivity to Anti-Mullerian Hormone Due to a Mutation in the Human Anti-Mullerian Hormone Receptor. Nat Genet (1995) 11(4):382–8. 10.1038/ng1295-382 7493017

[16] Clarke TR Hoshiya Y Yi SE Liu X Lyons KM Donahoe PK. Mullerian Inhibiting Substance Signalling Using a Bone Morphogenetic Pprotein (BMP)-Like Pathway Mediated by ALK2 and Induces SMAD6 Expression. Mol Endocrinol (2001) 15(6):946–59. 10.1210/mend.15.6.0664 11376113

[17] Gouedard L Chen YG Thevenet L Racine C Borie S Lamarre I. Engagement of Bone Morphogenetic Protein Type IB Receptor and Smad1 Signaling by Anti-Mullerian Hormone and its Type II Receptor. J Biol Chem (2000) 275(36):27973–8. 10.1074/jbc.M002704200 10854429

[18] Jamin SP Arango NA Mishina Y Hanks MC Behringer RR. Requirement of Bmpr1a for Mullerian Duct Regression During Male Sexual Development. Nat Genet (2002) 32(3):408–10. 10.1038/ng1003 12368913

[19] Sedes L Leclerc A Moindjie H Cate RL Picard JY di Clemente N. Anti-Mullerian Hormone Recruits BMPR-IA in Immature Granulosa Cells. PloS One (2013) 8(11):e81551. 10.1371/journal.pone.0081551 24312319 PMC3842941

[20] Ferlay J Ervik M Lam F Colombet M Mery L Piñeros M. Global Cancer Observatory: Cancer Today. Lyon, France: International Agency for Research on Cancer (2018).

[21] Roser M Rtichie H. Cancer Our World in Data2019 . Available at: https://ourworldindata.org/cancer#the-number-of-cancer-deaths-is-increasing-as-the-world-population-is-growing-and-aging.

[22] Australian Bureau of Statistics. Births, by Nuptiality, by Age of Mother: Australian Bureau of Statistics (2020). Available at: http://stat.data.abs.gov.au/Index.aspx?DataSetCode=BIRTHS_AGE_MOTHER.

[23] Office for National Statistics. Birth Characteristics in England and Wales 2018. United Kingdom: Office for National Statistics (2019).

[24] Mathews TJ Hamilton BE. Mean Age of Mother. Natl Vital Stat Rep (2002) 51(1):1–13. 12564162

[25] Mathews TJ Hamilton BE. Mean Age of Mothers is on the Rise: United States, 2000-2014. NCHS Data Brief (2016) (232):1–8. 26828319

[26] Fertility Indicators. EUROSTAT (2020). Available at: https://appsso.eurostat.ec.europa.eu/nui/submitViewTableAction.do.

[27] Roser M Ritchie H. Cancer 2015 [Updated November 2019 . Available at: https://ourworldindata.org/cancer#is-the-world-making-progress-against-cancer.

[28] Partridge AH Gelber S Peppercorn J Sampson E Knudsen K Laufer M. Web-Based Survey of Fertility Issues in Young Women With Breast Cancer. J Clin Oncol (2004) 22(20):4174–83. 10.1200/JCO.2004.01.159 15483028

[29] Ruddy KJ Gelber SI Tamimi RM Ginsburg ES Schapira L Come SE. Prospective Study of Fertility Concerns and Preservation Strategies in Young Women With Breast Cancer. J Clin Oncol (2014) 32(11):1151–6. 10.1200/JCO.2013.52.8877 PMC4164759 24567428

[30] Senkus E Gomez H Dirix L Jerusalem G Murray E Van Tienhoven G. Attitudes of Young Patients With Breast Cancer Toward Fertility Loss Related to Adjuvant Systemic Therapies. EORTC Study 10002 BIG 3-98. Psychooncology (2014) 23(2):173–82. 10.1002/pon.3384 24038775

[31] Thewes B Meiser B Rickard J Friedlander M. The Fertility- and Menopause-Related Information Needs of Younger Women With a Diagnosis of Breast Cancer: A Qualitative Study. Psychooncology (2003) 12(5):500–11. 10.1002/pon.685 12833562

[32] Chang HM Klausen C Leung PC. Antimullerian Hormone Inhibits Follicle-Stimulating Hormone-Induced Adenylyl Cyclase Activation, Aromatase Expression, and Estradiol Production in Human Granulosa-Lutein Cells. Fertil Steril (2013) 100(2):585–92 e1. 10.1016/j.fertnstert.2013.04.019 23663993

[33] Devillers MM Petit F Cluzet V François CM Giton F Garrel G. FSH Inhibits AMH to Support Ovarian Estradiol Synthesis in Infantile Mice. J Endocrinol (2019) 240(2):215–28. 10.1530/JOE-18-0313 30403655

[34] Grossman MP Nakajima ST Fallat ME Siow Y. Mullerian-Inhibiting Substance Inhibits Cytochrome P450 Aromatase Activity in Human Granulosa Lutein Cell Culture. Fertil Steril (2008) 89(5 Suppl):1364–70. 10.1016/j.fertnstert.2007.03.066 17517397

[35] Li Y Gao D Xu T Adur MK Zhang L Luo L. Anti-Mullerian Hormone Inhibits Luteinizing Hormone-Induced Androstenedione Synthesis in Porcine Theca Cells. Theriogenology (2020) 142:421–32. 10.1016/j.theriogenology.2019.10.037 31711705

[36] Sacchi S D'Ippolito G Sena P Marsella T Tagliasacchi D Maggi E. The Anti-Mullerian Hormone (AMH) Acts as a Gatekeeper of Ovarian Steroidogenesis Inhibiting the Granulosa Cell Response to Both FSH and LH. J Assist Reprod Genet (2016) 33(1):95–100. 10.1007/s10815-015-0615-y 26631403 PMC4717146

[37] Hayes E Kushnir V Ma X Biswas A Prizant H Gleicher N. Intra-Cellular Mechanism of Anti-Mullerian Hormone (AMH) in Regulation of Follicular Development. Mol Cell Endocrinol (2016) 433:56–65. 10.1016/j.mce.2016.05.019 27235859

[38] Pellatt L Rice S Dilaver N Heshri A Galea R Brincat M. Anti-Müllerian Hormone Reduces Follicle Sensitivity to Follicle-Stimulating Hormone in Human Granulosa Cells. Fertility Sterility (2011) 96(5):1246–51.e1. 10.1016/j.fertnstert.2011.08.015 21917251

[39] Rouiller-Fabre V Carmona S Merhi A Cate R Havert R Vigier B. Effect of Anti-Mullerian Hormone on Sertoli and Leydig Cell Functions in Fetal and Immature Rats. Endocrinology (1998) 139(3):1213–20. 10.1210/endo.139.3.5785 9492056

[40] Mukherjee A Park-Sarge OK Mayo KE. Gonadotropins Induce Rapid Phosphorylation of the 3'5'-Cyclic Adenosine Monophosphae Response Element Binding Protein in Ovarian Granulosa Cells. Endocrinology (1996) 137(8):3234–45. 10.1210/endo.137.8.8754745 8754745

[41] Vigier B Forest MG Eychenne B Bezard J Garrigou O Robel P. Anti-Mullerian Hormone Produces Endocrine Sex Reversal of Fetal Ovaries. Proc Natl Acad Sci USA (1989) 1989:86:3684–8. 10.1073/pnas.86.10.3684 PMC287204 2726747

[42] Prapa E Vasilaki A Dafopoulos K Katsiani E Georgoulias P Messini CI. Effect of Anti-Mullerian Hormone (AMH) and Bone Morphogenetic Protein 15 (BMP-15) on Steroidogenesis in Primary-Cultured Human Luteinizing Granulosa Cells Through Smad5 Signalling. J Assist Reprod Genet (2015) 32(7):1079–88. 10.1007/s10815-015-0494-2 PMC4531871 26003656

[43] Laurich VM Trbovich AM O'Neill FH Houk CP Sluss PM Payne AH. Mullerian Inhibiting Substance Blocks the Protein Kinase A-Induced Expression of Cytochrome P450 17a-Hydroxylase/C17-20 Lyase mRNA in a Mouse Leydig Cell Line Independent of cAMH Responsive Element Binding Protein Phosphorylation. Endocrinology (2002) 143:3351–60. 10.1210/en.2001-211352 12193547

[44] Sriraman V Niu E Matias JR Donahoe PK MacLaughlin DT Hardy MP. Mullerian Inhibiting Substance Inhibits Testosterone Synthesis in Adult Rats. J Andrology (2001) 22(5):750–8. 11545286

[45] Teixeira J Fynn-Thompson E Payne AH Donahoe PK. Mullerian-Inhibiting Substance Regulates Androgen Synthesis at the Transcriptional Level. Endocrinology (1999) 140(10):4732–8. 10.1210/endo.140.10.7075 10499532

[46] Trbovich AM Sluss PM Laurich VM O'Neill FH MacLaughlin DT Donahoe PK. Mullerian Inhibiting Substance Lowers Testosterone in Luteinizing Hormone-Stimulated Rodents. PNAS (2001) 98(6):3393–7. 10.1073/pnas.051632298 PMC30664 11248089

[47] Lyet L Louis F Forest MG Josso N Behringer RR Vigier B. Ontogeny of Reproductive Abnormalities Induced by Deregulation of Anti-Mullerian Hormone Expression in Transgenic Mice. Biol Reprod (1995) 52:444–54. 10.1095/biolreprod52.2.444 7711213

[48] Kano M Sosulski AE Zhang L Saatcioglu HD Wang D Nagykery N. AMH/MIS as a Contraceptive That Protects the Ovarian Reserve During Chemotherapy. Proc Natl Acad Sci USA (2017) 114(9):E1688–E97. 10.1073/pnas.1620729114 PMC5338508 28137855

[49] Racine C Rey R Forest MG Louis F Ferre A Huhtaniemi I. Receptors for Anti-Mu¨ Llerian Hormone on Leydig Cells are Responsible for its Effects on Steroidogenesis and Cell Differentiation. Proc Natl Acad Sci USA (1998) 95(2):594–9. 10.1073/pnas.95.2.594 PMC18465 9435237

[50] Selvaraj V Stocco DM Clark BJ. Current Knowledge on the Acute Regulation of Steroidogenesis. Biol Reprod (2018) 99(1):13–26. 10.1093/biolre/ioy102 29718098 PMC6044331

[51] Aksglaede L Sørensen K Boas M Mouritsen A Hagen CP Jensen RB. Changes in Anti-Müllerian Hormone (AMH) Throughout the Life Span: A Population-Based Study of 1027 Healthy Males From Birth (Cord Blood) to the Age of 69 Years. J Clin Endocrinol Metab (2010) 95(12):5357–64. 10.1210/jc.2010-1207 20843948

[52] Rey R Lordereau-Richard I Carel JC Barbet P Cate RL Roger M. Anti-Mullerian Hormone and Testosterone Serum Levels are Inversely Related During Normal and Precocious Pubertal Development. J Clin Endocrinol Metab (1993) 77(5):1220–6. 10.1210/jcem.77.5.8077315 8077315

[53] Baker ML Hutson JM. Serum Levels of Mullerian Inhibiting Substance in Boys Throughout Puberty and in the First Two Years of Life. J Clin Endocrinol Metab (1993) 76(1):245–7. 10.1210/jcem.76.1.8421093 8421093

[54] Lee MM Donahoe PK Hasegawa T Silverman B Crist GB Best S. Mullerian Inhibiting Substance in Humans: Normal Levels From Infancy to Adulthood. J Clin Endocrinol Metab (1996) 81:571–6. 10.1210/jcem.81.2.8636269 8636269

[55] Al-Attar L Noël K Dutertre M Belville C Forest MG Burgoyne PS. Hormonal and Cellular Regulation of Sertoli Cell Anti-Müllerian Hormone Production in the Postnatal Mouse. J Clin Invest (1997) 100(6):1335–43. 10.1172/JCI119653 PMC508311 9294098

[56] Josso N Legeai L Forest MG Chaussain JL Brauer PR. An Enzyme Linked Immunoassay for Anti-Müllerian HormoneL: A New Tool for the Evaluation of Testicular Function in Infants and Children. J Clin Endocrinol Metab (1990) 70(1):23–7. 10.1210/jcem-70-1-23 1688440

[57] Edelsztein NY Racine C di Clemente N Schteingart HF Rey RA. Androgens Downregulate Anti-Mullerian Hormone Promoter Activity in the Sertoli Cell Through the Androgen Receptor and Intact Steroidogenic Factor 1 Sites. Biol Reprod (2018) 99(6):1303–12. 10.1093/biolre/ioy152 29985989

[58] Durlinger AL Gruijters JG Kramer P Karels B Ingraham HA Nachtigal MW. Anti-Mullerian Hormone Inhibits Initiation of Primordial Follicle Growth in the Mouse Ovary. Endocrinology (2002) 143(3):1076–84. 10.1210/endo.143.3.8691 11861535

[59] Durlinger AL Gruijters JG Kramer P Karels B Kumar TR Matzuk MM. Anti-Mullerian Hormone Attenuates the Effects of FSH on Follicle Development in the Mouse Ovary. Endocrinology (2001) 142(11):4891–9. 10.1210/endo.142.11.8486 11606457

[60] Durlinger AL Kramer P Karels B de Jong FH Uilenbroek JTJ Grootegoed JA. Control of Primordial Follicle Recruitment by Anti-Mullerian Hormone in the Mouse Ovary. Endocrinology (1999) 140(12):5789–96. 10.1210/endo.140.12.7204 10579345

[61] Guo R Pankhurst MW. Accelerated Ovarian Reserve Depletion in Female Anti-Mullerian Hormone Knockout Mice has No Effect on Lifetime Fertilitydagger. Biol Reprod (2020) 102(4):915–22. 10.1093/biolre/ioz227 31837140

[62] Durlinger AL Visser JA Themmen AP. Regulation of Ovarian Function: The Role of Anti-MüLlerian Hormone. Reproduction (2002) 124:601–9. 10.1530/rep.0.1240601 12416998

[63] Carlsson IB Scott JE Visser JA Ritvos O Themmen AP Hovatta O. Anti-Mullerian Hormone Inhibits Initiation of Growth of Human Primordial Ovarian Follicles *In Vitro* . Hum Reprod (2006) 21(9):2223–7. 10.1093/humrep/del165 16720622

[64] Schmidt KL Kryger-Baggesen N Byskov AG Andersen CY. Anti-Mullerian Hormone Initiates Growth of Human Primordial Follicles *In Vitro* . Mol Cell Endocrinol (2005) 234(1-2):87–93. 10.1016/j.mce.2004.12.010 15836957

[65] Wright CS Hovatta O Margara R Trew G Winston RML Franks S. Effects of Follicle-Stimulating Hormone and Serum Substitution on the *in-Vitro* Growth of Human Ovarian Follicles. Hum Reprod (1999) 14(6):1552–62. 10.1093/humrep/14.6.1555 10357975

[66] McGee EA Smith R Spears N Nachtigal MW Ingraham HA Hsueh AJW. Mullerian Inhibitory Substance Induces Growth of Rat Preantral Ovarian Follicles. Biol Reprod (2001) 64:293–8. 10.1095/biolreprod64.1.293 11133686

[67] Xu J Bishop CV Lawson MS Park BS Xu F. Anti-Mullerian Hormone Promotes Pre-Antral Follicle Growth, But Inhibits Antral Follicle Maturation and Dominant Follicle Selection in Primates. Hum Reprod (2016) 31(7):1522–30. 10.1093/humrep/dew100 PMC4901882 27165618

[68] Bedoschi G Navarro PA Oktay K. Chemotherapy-Induced Damage to Ovary: Mechanisms and Clinical Impact. Future Oncol (2016) 12(20):2333–44. 10.2217/fon-2016-0176 PMC5066134 27402553

[69] Soleimani R Heytens E Darzynkiewicz Z Oktay K. Mechanisms of Chemotherapy-Induced Human Ovarian Aging- Double Strand DNA Breaks and Microvascular Compromise. Aging (2011) 3(8):1–12. 10.18632/aging.100363 21869459 PMC3184979

[70] Meirow D Dor J Kaufman B Shrim A Rabinovici J Schiff E. Cortical Fibrosis and Blood-Vessels Damage in Human Ovaries Exposed to Chemotherapy. Potential Mechanisms of Ovarian Injury. Hum Reprod (2007) 22(6):1626–33. 10.1093/humrep/dem027 17324957

[71] Marcello MF Nuciforo G Romeo R di Dono G Russo I Russo A. Structural and Ultrastructural Study of the Ovary in Childhood Leukemia After Successful Treatment. Cancer (1990) 66(10):2099–104. 10.1002/1097-0142(19901115)66:10<2099::AID-CNCR2820661010>3.0.CO;2-3 2224764

[72] Plowchalk DR Mattison DR. Reproductive Toxicity of Cyclophosphamide in the C57BL/6N Mouse: Effects on Ovarian Structure and Function. Reprod Toxicol (1992) 6:411–21. 10.1016/0890-6238(92)90004-D 1463921

[73] Ataya KM Valeriote FA Ramahi-Ataya AJ. Effect of Cyclophosphamide on the Immature Rat Ovary. Cancer Res (1989) 49:1660–4. 2924314

[74] Lopez SG Luderer U. Effects of Cyclophosphamide and Buthionine Sulfoximine on Ovarian Glutathione and Apoptosis. Free Radic Biol Med (2004) 36(11):1366–77. 10.1016/j.freeradbiomed.2004.02.067 15135172

[75] Davis BJ Heindel JJ. Ovarian Toxicants: Multiple Mechanisms of Action. In: Korach KS, editor. Reproductive and Developmental Toxicology. New York: Dekker (1998). p. 373–95.

[76] Badawy A Elnashar A El-Ashry M Shahat M. Gonadotropin-Releasing Hormone Agonists for Prevention of Chemotherapy-Induced Ovarian Damage: Prospective Randomized Study. Fertility Sterility (2009) 91(3):694–7. 10.1016/j.fertnstert.2007.12.044 18675959

[77] Chen H Li J Cui T Hu L. Adjuvant Gonadotropin-Releasing Hormone Analogues for the Prevention of Chemotherapy Induced Premature Ovarian Failure in Premenopausal Women. Cochrane Database Systematic Rev (2011) 11. 10.1002/14651858.CD008018.pub2 22071842

[78] Del Mastro L Ceppi M Poggio F Bighin C Peccatori F Demeestere I. Gonadotropin-Releasing Hormone Analogues for the Prevention of Chemotherapy-Induced Premature Ovarian Failure in Cancer Women: Systematic Review and Meta-Analysis of Randomized Trials. Cancer Treat Rev (2014) 40(5):675–83. 10.1016/j.ctrv.2013.12.001 24360817

[79] Elgindy EA El-Haieg DO Khorshid OM Ismail EI Abdelgawad M Sallam HN. Gonadatrophin Suppression to Prevent Chemotherapy-Induced Ovarian Damage: A Randomized Controlled Trial. Obstet Gynecol (2013) 121(1):78–86. 10.1097/AOG.0b013e31827374e2 23262931

[80] Mehta RR Beattie CW Das Gupta TK. Endocrine Profile in Breast Cancer Patients Receiving Chemotherapy. Breast Cancer Res Treat (1991) 20:125–32. 10.1007/BF01834642 1554888

[81] Oktay K Briggs D Gosden RG. Ontogeny of Follicle-Stimulating Hormone Receptor Gene Expression in Isolated Human Ovarian Follicles. J Clin Endocrinol Metab (1997) 82(11):3748–51. 10.1210/jc.82.11.3748 9360535

[82] Kalich-Philosoph L Roness H Carmely A Fishel-Bartal M Ligumsky H Paglin S. Cyclophosphamide Triggers Follicle Activation and Burnout; AS101 Prevents Follicle Loss and Preserves Fertility. Sci Transl Med (2013) 5(185):1–11. 10.1126/scitranslmed.3005402 23677591

[83] Sonigo C Beau I Grynberg M Binart N. AMH Prevents Primordial Ovarian Follicle Loss and Fertility Alteration in Cyclophosphamide-Treated Mice. FASEB J (2019) 33(1):1278–87. 10.1096/fj.201801089R 30113879

[84] Roness H Spector I Leichtmann-Bardoogo Y Savino AM Dereh-Haim S Meirow D. Pharmacological Administration of Recombinant Human AMH Rescues Ovarian Reserve and Preserves Fertility in a Mouse Model of Chemotherapy, Without Interfering With Anti-Tumoural Effects. J Assist Reprod Genet (2019) 36(9):1793–803. 10.1007/s10815-019-01507-9 PMC6730972 31250176

[85] Weenen C Laven JS Von Bergh AR Cranfield M Groome NP Visser JA. Anti-Mullerian Hormone Expression Pattern in the Human Ovary: Potential Implications for Initial and Cyclic Follicle Recruitment. Mol Hum Reprod (2004) 10(2):77–83. 10.1093/molehr/gah015 14742691

[86] Donnez J Dolmans MM. Fertility Preservation in Women. N Engl J Med (2017) 377(17):1657–65. 10.1056/NEJMra1614676 29069558

[87] Dolmans MM Martinez-Madrid B Gadisseux E Guiot Y Yuan WY Torre A. Short-Term Transplantation of Isolated Human Ovarian Follicles and Cortical Tissue Into Nude Mice. Reproduction (2007) 134(2):253–62. 10.1530/REP-07-0131 17660235

[88] Liu J van der Elst J van den Broecke R Dhont M. Early Massive Follicle Loss and Apoptosis in Heterotropically Grafted Newborn Mouse Ovaries. Hum Reprod (2002) 17(2):605–11. 10.1093/humrep/17.3.605 11870111

[89] Gavish Z Spector I Peer G Schlatt S Wistuba J Roness H. Follicle Activation is a Significant and Immediate Cause of Follicle Loss After Ovarian Tissue Transplantation. J Assist Reprod Genet (2018) 35:61–9. 10.1007/s10815-017-1079-z PMC5758475 29098533

[90] Donnez J Dolmans MM Diaz C Pellicer A. Ovarian Cortex Transplantation: Time to Move on From Experimental Studies to Open Clinical Application. Fertil Steril (2015) 104(5):1097–8. 10.1016/j.fertnstert.2015.08.005 26342246

[91] Van der Ven H Liebenthron J Beckmann M Toth B Korell M Krussel J. Ninety-Five Orthotopic Transplantations in 74 Women of Ovarian Tissue After Cytotoxic Treatment in a Fertility Preservation Network: Tissue Activity, Pregnancy and Delivery Rates. Hum Reprod (2016) 31(9):2031–41. 10.1093/humrep/dew165 27378768

[92] Fortune JE Cushman RA Wahl CM Kito S. The Primordial to Primary Follicle Transition. Mol Cell Endocrinol (2000) 163:53–60. 10.1016/S0303-7207(99)00240-3 10963874

[93] Braw-Tal R Yossefi S. Studies *In Vivo* and *In Vitro* on the Initiation of Follicle Growth in the Bovine Ovary. J Reprod Fertility (1997) 109(1):165–71. 10.1530/jrf.0.1090165 9068428

[94] Lass A Silye R Abrams DC Krausz T Hovatta O Margara R. Follicular Density in Ovarian Biopsy of Infertile Women- a Novel Method to Assess Ovarian Reserve. Hum Reprod (1997) 12(5):1028–31. 10.1093/humrep/12.5.1028 9194660

[95] Ayuandari S Winkler-Crepaz K Paulitsch M Wagner C Zavadil C Manzl C. Follicular Growth After Xenotransplantation of Cryopreserved/Thawed Human Ovarian Tissue in SCID Mice: Dynamics and Molecular Aspects. J Assist Reprod Genet (2016) 33(12):1585–93. 10.1007/s10815-016-0769-2 PMC5171895 27465301

[96] Silber S Pineda J Lenahan K DeRosa M Melnick J. Fresh and Cryopreserved Ovary Transplantation and Resting Follicle Recruitment. Reprod BioMed Online (2015) 30(6):643–50. 10.1016/j.rbmo.2015.02.010 25892498

[97] Kong HS Kim SK Lee J Youm HW Lee JR Suh CS. Effect of Exogenous Anti-Müllerian Hormone Treatment on Cryopreserved and Transplanted Mouse Ovaries. Reprod Sci (2016) 23(1):51–60. 26156852 10.1177/1933719115594021

[98] Vigier B Tran D du Mesnil du Buisson F Heyman Y Josso N. Use of Monoclonal Antibody Techniques to Study the Ontogeny of Bovine Anti-Müllerian Hormone. J Reprod Fertility (1983) 69(1):207–14. 10.1530/jrf.0.0690207 6350571

[99] Griesinger G Dafopoulos K Buendgen N Cascorbi I Georgoulias P Zavos A. Elimination Half-Life of Anti-Mullerian Hormone. J Clin Endocrinol Metab (2012) 97(6):2160–3. 10.1210/jc.2012-1070 22442264

[100] Donahoe PK Swann DA Hayashi A Sullivan MD. Mullerian Duct Regression in the Embryo Correlated With Cytotoxic Activity Against Human Ovarian Cancer. Science (1979) 205(4409):913–5. 10.1126/science.472712 472712

[101] Barbie TU Barbie DA MacLaughlin DT Maheswaran S Donahoe PK. Mullerian Inhibiting Substance Inhibits Cervical Cancer Cell Growth via a Pathway Involving P130 and P107. PNAS (2003) 100(26):15601–6. 10.1073/pnas.2636900100 PMC307614 14671316

[102] Hwang SJ Suh MJ Yoon JH Kim MR Ryu KS Nam SW. Identification of Characteristic Molecular Signature of Mullerian Inhibiting Substance in Human HPV-Related Cervical Cancer Cells. Int J Oncol (2011) 39(4):811–20. 10.3892/ijo.2011.1042 PMC5609187 21573503

[103] Fuller AF Budzik IM Donahoe PK. Mullerian Inhibiting Substance Inhibition of a Human Endometrial Carcinoma Cell Line Xenografted in Nude Mice. Gynecol Oncol (1984) 17:124–32. 10.1016/0090-8258(84)90066-0 6546372

[104] Renaud EJ MacLaughlin DT Oliva E Rueda BR Donahoe PK. Endometrial Cancer is a Receptor-Mediated Target for Mullerian Inhibiting Substance. Proc Natl Acad Sci USA (2005) 102(1):111–6. 10.1073/pnas.0407772101 PMC544070 15618407

[105] Gupta V Carey JL Kawakubo H Muzikansky A Green JE Donahoe PK. Mullerian Inhibiting Substance Suppresses Tumor Growth in the C3(1)T Antigen Transgenic Mouse Mammary Carcinoma Model. Proc Natl Acad Sci USA (2005) 102(9):3219–24. 10.1073/pnas.0409709102 PMC552936 15728372

[106] Hoshiya Y Gupta V Segev DL Hoshiya M Carey JL Sasur LM. Mullerian Inhibiting Substance Induces NFkB Signaling in Breast and Prostate Cancer Cells. Mol Cell Endocrinol (2003) 211(1-2):43–9. 10.1016/j.mce.2003.09.010 14656475

[107] Segev DL Ha TU Tran TT Kenneally M Harkin P Jung M. Mullerian Inhibiting Substance Inhibits Breast Cancer Cell Growth Through an NFkappa B-Mediated Pathway. J Biol Chem (2000) 275(37):28371–9. 10.1074/jbc.M004554200 10874041

[108] Chin T Parry RL Donahoe PK. Human Mullerian Inhibiting Substance Inhibits Tumour Growth *In Vitro* . Cancer Res (1991) 51:2101–6. 2009529

[109] Dubeau L. The Cell of Origin of Ovarian Epithelial Tumours. Lancet Oncol (2008) 9(12):1191–7. 10.1016/S1470-2045(08)70308-5 PMC4176875 19038766

[110] Kurman RJ Shih Ie M. Molecular Pathogenesis and Extraovarian Origin of Epithelial Ovarian Cancer–Shifting the Paradigm. Hum Pathol (2011) 42(7):918–31. 10.1016/j.humpath.2011.03.003 PMC3148026 21683865

[111] Vang R Shih Ie M Kurman RJ. Fallopian Tube Precursors of Ovarian Low- and High-Grade Serous Neoplasms. Histopathology (2013) 62(1):44–58. 10.1111/his.12046 23240669

[112] Anttonen M Farkkila A Tauriala H Kauppinen M Maclaughlin DT Unkila-Kallio L. Anti-Mullerian Hormone Inhibits Growth of AMH Type II Receptor-Positive Human Ovarian Granulosa Cell Tumor Cells by Activating Apoptosis. Lab Invest (2011) 91(11):1605–14. 10.1038/labinvest.2011.116 21808236

[113] Masiakos PT MacLaughlin DT Maheswaran S Teixeira J Fuller AF Shah PC. Human Ovarian Cancer, Cell Lines and Primary Ascites Cells Express the Human Mullerian Inhibiting Substance (MIS) Type II Receptor, Bind, and are Responsive to MIS. Clin Cancer Res (1999) 5:3488–99. 10589763

[114] Donahoe PK Fuller AF Scully RE Guy SR Budzik GP. Mullerian Inhibiting Substance Inhibits Growth of a Human Ovarian Cancer in Nude Mice. Ann Surg (1981) 194(4):472–80. 10.1097/00000658-198110000-00010 PMC1345325 6895157

[115] Fuller AF Krane IM Budzik GP Donahoe PK. Mullerian Inhibiting Substance Reduction of Colony Growth of Human Gynecologic Cancers in a Stem Cell Assay. Gynecol Oncol (1985) 22:135–48. 10.1016/0090-8258(85)90019-8 3932140

[116] Jung YS Kim HJ Seo SK Choi YS Nam EJ Kim S. Anti-Proliferative and Apoptotic Activities of Mullerian Inhibiting Substance Combined With Calcitriol in Ovarian Cancer Cell Lines. Yonsei Med J (2016) 57(1):33–40. 10.3349/ymj.2016.57.1.33 26632380 PMC4696969

[117] Pieretti-Vanmarcke R Donahoe PK Szotek P Manganaro T Lorenzen MK Lorenzen J. Recombinant Human Mullerian Inhibiting Substance Inhibits Long-Term Growth of MIS Type II Receptor-Directed Transgenic Mouse Ovarian Cancers *In Vivo* . Clin Cancer Res (2006) 12(5):1593–8. 10.1158/1078-0432.CCR-05-2108 16533786

[118] Stephen AE Pearsall LA Christian BP Donahoe PK Vacanti JP MacLaughlin DT. Highly Purified Mullerian Inhibiting Substance Inhibits Human Ovarian Cancer *In Vivo* . Clin Cancer Res (2002) 8:2640–6. 12171896

[119] Chang HL Pieretti-Vanmarcke R Nicolaou F Li X Wei X MacLaughlin DT. Mullerian Inhibiting Substance Inhibits Invasion and Migration of Epithelial Cancer Cell Lines. Gynecol Oncol (2011) 120(1):128–34. 10.1016/j.ygyno.2010.09.017 PMC3008816 21056908

[120] Davidson B. Recently Identified Drug Resistance Biomarkers in Ovarian Cancer. Expert Rev Mol Diagn (2016) 16(5):569–78. 10.1586/14737159.2016.1156532 26895188

[121] Chen K Huang YH Chen JL. Understanding and Targeting Cancer Stem Cells: Therapeutic Implications and Challenges. Acta Pharmacol Sin (2013) 34(6):732–40. 10.1038/aps.2013.27 PMC3674516 23685952

[122] Lupia M Cavallaro U. Ovarian Cancer Stem Cells: Still an Elusive Entity? Mol Cancer (2017) 16(1):64. 10.1186/s12943-017-0638-3 28320418 PMC5360065

[123] Wei X Dombkowski DM Meirelles K Pieretti-Vanmarcke R Szotek P Chang HL. Mullerian Inhibiting Substance Preferentially Inhibits Stem/Progenitors in Human Ovarian Cancer Cell Lines Compared With Chemotherapeutics. PNAS (2010) 107(44):18874–9. 10.1073/pnas.1012667107 PMC2973919 20952655

[124] Meirelles K Benedict LA Dombkowski DM Pepin D Preffer FI Teixeira J. Human Ovarian Cancer Stem/Progenitor Cells are Stimulated by Doxorubicin But Inhibited by Mullerian Inhibiting Substance. PNAS (2012) 109(7):2358–63. 10.1073/pnas.1120733109 PMC3289387 22308459

[125] Pepin D Sosulski A Zhang L Wang D Vathipadiekal V Hendren K. AAV9 Delivering a Modified Human Mullerian Inhibiting Substance as a Gene Therapy in Patient-Derived Xenografts of Ovarian Cancer. Proc Natl Acad Sci USA (2015) 112(32):E4418–27. 10.1073/pnas.1510604112 PMC4538667 26216943

[126] Kersual N Garambois V Chardes T Pouget JP Salhi I Bascoul-Mollevi C. The Human Mullerian Inhibiting Substance Type II Receptor as Immunotherapy Target for Ovarian Cancer. Validation Using the mAb 12g4. MAbs (2014) 6(5):1314–26. 10.4161/mabs.29316 PMC4623115 25517316

[127] Herter S Birk MC Klein C Gerdes C Umana P Bacac M. Glycoengineering of Therapeutic Antibodies Enhances Monocyte/Macrophage-Mediated Phagocytosis and Cytotoxicity. J Immunol (2014) 192(5):2252–60. 10.4049/jimmunol.1301249 PMC3932809 24489098

[128] Prat M Salon M Allain T Dubreuil O Noel G Preisser L. Murlentamab, a Low Fucosylated Anti-Mullerian Hormone Type II Receptor (AMHRII) Antibody, Exhibits Anti-Tumor Activity Through Tumor-Associated Macrophage Reprogrammation and T Cell Activation. Cancers (Basel) (2021) 13(8):1845. 10.3390/cancers13081845 33924378 PMC8070390

[129] Leary A Awada A Delord JP Floquet A Ray-Coquard IL Abdeddaim C. First-In-Human First-in-Class Phase I Trial of Murlentamab, an Anti-Mullerian-Hormone Receptor II (AMHRII) Monoclonal Antibody Acting Through Tumor-Associated Macrophage (TAM) Engagement, as Single Agent and in Combination With Carboplatin (C) and Paclitaxel (P) in AMHRII-Expressing Advanced/Metastatic Gynecological Cancer Patients (Pts). J Clin Oncol (2019) 37(15s):2521–. 10.1200/JCO.2019.37.15suppl.2521

[130] van Cutsem E Melichar B van den Eynde M Prausova J Geboes K Dekervel J. Phase 2 Study Results of Murlentamab, a Monoclonal Antibody Targeting the Anti-Mullerian-Hormone-Receptor II (AMHRII), Acting Through Tumor-Associated Macrophage Engagement Inadvanced/Metastatic Colorectal Cancers. Ann Oncol (2019) 30(S4):iv153–iv4. 10.1093/annonc/mdz183.003

[131] Ladjohounlou R Pichard A Dehayes E Boudousq V Bruchertseifer F Morgenstern A. Therapeutic Efficacy of Brief Intraperitoneal Radioimmunotherapy of Ovarian Cancer Using 213Bi-Anti MISRII Antibodies. Eur J Nucl Med Mol Imaging (2016) 43(Suppl 1):1–734. 10.2967/jnumed.118.208611

[132] Ortega C Herbet A Richard S Kersual N Costa N Pelegrin A. High Level Prokaryotic Expression of Anti-Mullerian Inhibiting Substance Type II Receptor Diabody, a New Recombinant Antibody for *In Vivo* Ovarian Cancer Imaging. J Immunol Methods (2013) 387(1-2):11–20. 10.1016/j.jim.2012.08.003 22910001

[133] Tal R Seifer DB Khanimov M Grazi RV Leader B. Characterization of Women With Ultra High Serum Anti-Mullerian Hormone (AMH) Levels and its Association With Polycystic Ovarian Syndrome (PCOS) Phenotype. Fertility Sterility (2013) 100(3):S127. 10.1016/j.fertnstert.2013.07.1610

[134] Chang HL Pahlavan N Halpern EF MacLaughlin DT. Serum Mullerian Inhibiting Substance/anti-Mullerian Hormone Levels in Patients With Adult Granulosa Cell Tumors Directly Correlate With Aggregate Tumor Mass as Determined by Pathology or Radiology. Gynecol Oncol (2009) 114(1):57–60. 10.1016/j.ygyno.2009.02.023 19359032 PMC2756071

[135] Torre LA Trabert B DeSantis CE Miller KD Samimi G Runowicz CD. Ovarian Cancer Statistics, 2018. CA Cancer J Clin (2018) 68(4):284–96. 10.3322/caac.21456 PMC6621554 29809280

